# Scleroderma and interstitial lung disease - A case report

**DOI:** 10.1016/j.amsu.2022.104143

**Published:** 2022-07-09

**Authors:** Fahad Gul, Amna Siddiqui, Prakhyath Srikaram, Nabeela Fatima

**Affiliations:** a Rawalpindi Medical University, Rawalpindi, Pakistan; b Karachi Medical and Dental College, Karachi City, Sindh, 74700, Pakistan; c Kurnool Medical College, Kurnool, 518002, Andhra Pradesh, India; d St Pauls College of Pharmacy, Turkayamjal, 501510, Hyderabad, India

**Keywords:** Scleroderma, Interstitial lung disease, Systemic sclerosis

## Abstract

**Background:**

Systemic sclerosis with interstitial lung disease is one of the rarely reported autoimmune disorders. The ILD associated with systemic sclerosis is the most common cause of mortality in these patients.

**Case presentation:**

A 37-year-old female patient who is a known case of Scleroderma, Cor pulmonale, and hypothyroidism presented with the exacerbated symptoms of dyspnea and orthopnea. On examination, she had digital gangrene as a dermatological complication of systemic sclerosis. The patient was given medical management and was improving.

**Discussion:**

ILD is the dreaded complication of systemic sclerosis. Pulmonary hypertension that developed secondary to the ILD in this patient led to the cor pulmonale. The patient has the exacerbation of the same.

**Conclusion:**

Early detection and management of the ILD-SS are very important to prevent progression, exacerbations, and morbidity associated with it.

## Highlights

• Scleroderma patients can present with exacerbation of existing conditions like interstitial lung disease.
• Early recognition and management of such cases can prevent morbidity and mortality.
• The ILD associated with systemic sclerosis is the most common cause of mortality in these patients.

## 1 Introduction

A chronic autoimmune disease characterized by thickening and fibrosis of the skin is known as Systemic sclerosis or scleroderma (SSc). It is a connective tissue disorder of unknown etiology, with variable clinical manifestations, chronic and usually a progressive course, which is often presented with multi-organ involvement, including lungs [1]. Lung fibrosis occurs in approximately four-fifths of patients with SSc; approximately one-fourth develop progressive interstitial lung disease (ILD), with 10-year mortality of around 40%, making it one of the leading causes of morbidity and mortality [2,3].

Since, SSc is a rare disease across the globe, as well as in the Asian population and thus, might be overlooked in clinical practice [4,5]. Its early diagnosis, is often a source of challenge for medical practitioners, due to its early presentation comprising of, non-specific clinical manifestations such as cough, dyspnea, and chest pain [6].

Early diagnosis of SSc-ILD is crucial to initiate treatment and prevent disease progression, thus, High-resolution computed tomography (HRCT) of the chest is recognized as a modality of choice for diagnosing and assessing SSc-ILD [7]. Further, the latter demands familiarity with HRCT findings and thorough clinical examination.

This case report has been reported in line with the SCARE 2020 guidelines [9]. To the best of our knowledge, this is the first detailed case report describing the clinical presentation and HRCT findings of a patient with SSc-ILD in the country. This case aims to raise awareness of this rare entity and emphasize the need for its early detection, strict management, and the utter need for the development of safe and effective treatments, that are capable of improving outcomes and disease progression.

## 2 Case presentation

A 37-year-old female patient with known complaints of Scleroderma associated with ILD, Cor pulmonale, and hypothyroidism presented to the hospital via OPD with complaints of SOB Grade-IV and orthopnea, which was not accompanied by wheeze. She had a history of cough and pain in the fingers of both hands, one year back. Over the last 10 days, the patient developed breathlessness. Gradually patient complained of loss of wrinkling over the face and limitation of movement at finger joints due to skin tightening, accompanied by pain in the digits. The patient's drug history, allergy history, family history, and psychosocial history were not significant.

**Fig-1:**
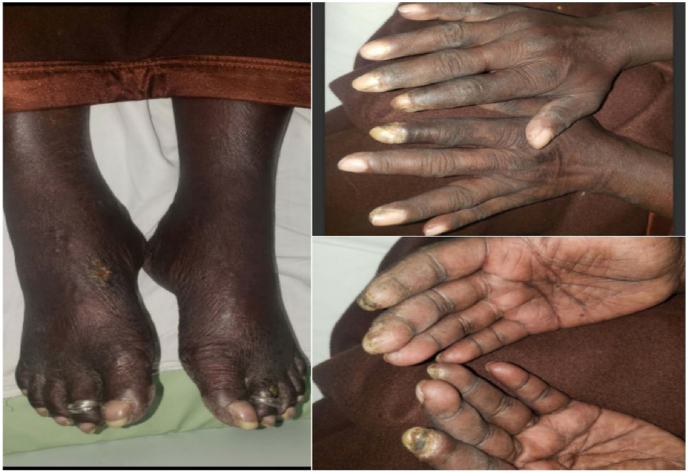
Dermatologic findings.

On inspection, findings were, diffuse skin pigmentation, with particularly salt and pepper pigmentation found in the face and neck region, impending digital gangrene of, the index fingers bilaterally and an ulcer noted on the right foot (Fig. 1). On general and systemic examination, findings were unremarkable, except in RS findings, bilateral crept were heard.

Moving forward, she had a history of nail removal of the index finger one week back and developed wounds in the area that were progressive. On further evaluation, the patient is on a home oxygenator. The 2D echocardiography showed pulmonary hypertension with a right atrium and ventricular dilation accompanied by severe tricuspid regurgitation. High Resolution Computed Tomography (HRCT) of the chest was done which revealed decreased volume of both lungs with diffuse ground attenuation and traction bronchiectasis and bronchiectasis in bilateral lung fields. Notably, subpleural interlobular and intralobular septal thickening with increased reticulation was found, along with focal areas of early honeycombing-that is suggestive of ILD-fibrotic non-specific interstitial pneumonia (NSIP) pattern. Hepatomegaly with an elevated dome of the diaphragm was also spotted.

The diagnosis is made based on his findings of bilateral thickening of fingers on both hands extending up to metacarpophalangeal joints as per the 2013 American College of Rheumatology (ACR) criteria. Conclusively, the above findings gave us a diagnosis of diffuse scleroderma with ILD-exacerbation.

The patient was treated with Inj. Cefpirome 1g IV BD, Tab.Levocetrizine + Monteleukast, Tab. Torsemide, Tab. Sildenafil, Tab.Mycophenolate 500mg PO BD, Tab.Hydrocortisone 50mg IV BD, Tab.Aspirin, Tab. Ambrisentan, Tab. Gabapentin 100ng PO OD, Tab.Prazosin 1mg PO BD during the hospital stay. She showed significant improvement and was discharged in stable condition.

## 3 Discussion

**Fig-2:**
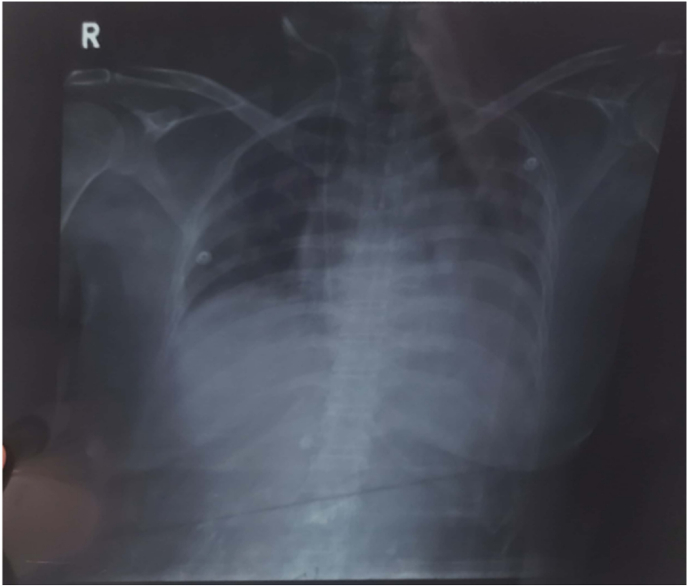
X-ray findings.

Our patient presented with acute exacerbation of symptoms of scleroderma-associated interstitial lung disease complicated by cor pulmonale. CT chest has shown ground attenuation, traction bronchiectasis, septal thickening, and honeycombing which are consistent with standard findings of interstitial lung disease in HRCT which is considered the most sensitive and gold standard modality in the diagnosis of SSc-ILD and has the utmost importance in monitoring disease prognosis. Despite established guidelines to use HRCT for SSc-ILD diagnosis only 66% of SSc experts are using it. Lack of clinical experience, lack of adequate knowledge, radiation exposure, imaging cost, and feasibility may be among the factors in its hindrance [2]. Chest x-ray AP view showed characteristic patchy consolidation in the left mid and lower lobe with ill-defined left costophrenic angle and left dome of diaphragm-effusion while right costophrenic angle and right dome of the diaphragm were normal which depicted that the left lung was more involved than the right one in our patient (Fig. 2).

Skin involvement is one of the earliest and most frequent manifestations of scleroderma, so patients encounter dermatologists as first-line doctors. Consistent with this criterion our patient presented with salt and pepper pigmentation of the face and neck, diffuse skin pigmentation, and bilateral index finger gangrene representing the advanced stage cutaneous manifestation.

The patient also developed pulmonary hypertension secondary to interstitial lung disease. Pulmonary hypertension had progressed to such an extent to cause right heart failure in this patient. This is called cor pulmonale. This can be evidenced by the 2D Echo showing the findings of the right atrium and ventricular dilation with severe tricuspid regurgitation.

Management of the ILD is mainly with corticosteroids. Symptomatic management is warranted if the patient has associated conditions. The literature recommends the usage of Mycophenolate mofetil in the management of SS-ILD in addition to corticosteroids [8]. The patient was put on medications for pulmonary hypertension like Ambrisenton, an endothelin antagonist which inhibits vasoconstriction, and sildenafil, a PDE5 inhibitor that promotes vasodilation, especially in pulmonary arteries. The prompt management of pulmonary hypertension treats the cor pulmonale and its exacerbations subsequently. The patient was also put on torsemide and spironolactone which is a combination of loop diuretic and potassium-sparing diuretic to treat fluid backup in cor pulmonale. The patient has a satisfactory course in the hospitalization and was discharged with almost the same medications and routine follow-up.

## 4 Conclusion

A 37-year-old female is diagnosed with systemic sclerosis - ILD. The gold standard non-invasive diagnosis can be made with HRCT which can record even the early changes. Management is mainly with corticosteroids and supportive medications. Early detection and prompt management are needed to prevent morbidity and mortality.

## Sources of funding for your research

None.

## Ethical approval

Ethical approval was not required as per country guidelines.

## Consent

Written informed consent was obtained from the patient for publication of this case report and accompanying images. A copy of the written consent is available for review by the Editor-in-Chief of this journal on request.

## Author contribution

Fahad Gul: Study concept, data collection, interpretation, manuscript writing, Amna Siddiqui: Study concept, data collection, interpretation, manuscript writing, Prakhyath Srikaram: Study concept, Interpretation, manuscript writing, Dr. Nabeela Fatima: Study concept, data collection, interpretation, manuscript writing.

## Registration of research studies

1Name of the registry: Not Applicable
2 Unique Identifying number or registration ID: Not Applicable
3 Hyperlink to your specific registration (must be publicly accessible and will be checked): Not Applicable

## Guarantor

Dr. Nabeela Fatima.

## Provenance and peer review

Not commissioned, externally peer-reviewed.

## Declaration of competing interest

No conflict of interest to be declared.
